# Exploiting fast detectors to enter a new dimension in room-temperature crystallography

**DOI:** 10.1107/S1399004714005379

**Published:** 2014-04-26

**Authors:** Robin L. Owen, Neil Paterson, Danny Axford, Jun Aishima, Clemens Schulze-Briese, Jingshan Ren, Elizabeth E. Fry, David I. Stuart, Gwyndaf Evans

**Affiliations:** a Diamond Light Source, Harwell Science and Innovation Campus, Didcot OX11 0DE, England; b Dectris Ltd, Neuenhofer Strasse 107, 5400 Baden, Switzerland; c Division of Structural Biology, University of Oxford, The Henry Wellcome Building for Genomic Medicine, Roosevelt Drive, Oxford OX3 7BN, England

**Keywords:** radiation damage, room temperature, macromolecular crystallography, dose rate

## Abstract

A departure from a linear or an exponential intensity decay in the diffracting power of protein crystals as a function of absorbed dose is reported. The observation of a lag phase raises the possibility of collecting significantly more data from crystals held at room temperature before an intolerable intensity decay is reached. A simple model accounting for the form of the intensity decay is reintroduced and is applied for the first time to high frame-rate room-temperature data collection.

## Abstract 2

A departure from a linear or an exponential decay in the diffracting power of macromolecular crystals is observed and accounted for through consideration of a multi-state sequential model.

## 1. Introduction   

Room-temperature data collection remains an important tool in macromolecular crystallography (MX), despite the dominance of cryocrystallography. Cryoprotection and the subsequent cooling process can damage samples, making the assessment of crystallization conditions difficult or even impossible. Furthermore, cryocooling can also hide conformational diversity (Fraser *et al.*, 2011 ▶) or introduce artefacts in side-chain interactions (Juers & Matthews, 2004 ▶), making a room-temperature structure desirable even in cases where samples prove amenable to cryocooling.

New facilities for *in situ* crystallography such as those described by Jacquamet *et al.* (2004 ▶), Bingel-Erlenmeyer *et al.* (2011 ▶) and Axford *et al.* (2012 ▶) have aided a renaissance in room-temperature MX at synchrotron sites, removing the need for crystal manipulation by allowing a crystallization tray to be mounted directly in the X-ray beam. The concomitant development of crystallization trays, chips and other hardware has resulted in simplified experimental setups and reduced diffuse scatter, and has furthered the uptake of the method (Soliman *et al.*, 2011 ▶; Kisselman *et al.*, 2011 ▶) so that useful biological results can now be readily obtained (see, for instance, Wang *et al.*, 2012 ▶; Porta *et al.*, 2013 ▶).

Despite these advances a fundamental challenge remains: the seemingly inevitable rapid onset of radiation damage means that the amount of data that can be collected from room-temperature crystals is severely limited. This impacts both crystal screening and structure solution and is the primary reason why data collection at room temperature is not routine at all synchrotron sources. In the case of crystal screening the rapid onset of radiation damage limits the use of tools such as the diffraction grid scan (Aishima *et al.*, 2010 ▶; Bowler *et al.*, 2010 ▶; Cherezov *et al.*, 2009 ▶) to find and assess crystals, and precludes diffraction-based screening of room-temperature crystals prior to cryocooling. *In situ* crystallo­graphy is by no means limited to crystal screening, however. Some samples, most notably viruses, can prove virtually impossible to successfully cryocool, meaning that all data must be collected at room temperature, and, as noted above, a physiologically more relevant structure determined at room temperature may provide additional insight or confirmation of the absence of cooling-induced artefacts.

For room-temperature structure solution, radiation damage usually severely limits the amount of data that can be collected from each sample. This generally imposes the need for a large number of (isomorphous and well diffracting) crystals, which in turn can make subsequent steps in the structure-solution pipeline such as data integration, scaling and phasing more challenging. Increasing the amount of data that can be collected from crystals by changing the manner of data collection would alleviate these problems and allow useful data to be collected from more weakly diffracting crystals.

In contrast to 100 K, where little or no dose-rate effect has been observed (Sliz *et al.*, 2003 ▶; Garman, 2010 ▶), at room temperature the rate at which data are collected can have a profound effect on the volume of data that it is possible to obtain. Recent experiments have revealed a systematic, and significant, increase in the dose tolerance of room-temperature protein and virus crystals as a function of dose rate (Owen *et al.*, 2012 ▶; Warkentin *et al.*, 2011 ▶, 2012 ▶). The collection of diffraction data continuously and at dose rates in excess of ∼0.7 MGy s^−1^ allowed more data to be collected from each crystal. In contrast, other recent studies (for example, Warkentin *et al.*, 2013 ▶; Leal *et al.*, 2013 ▶) conducted at lower dose rates have reported little dose-rate dependence at room temperature.

A dose-rate-dependent increase in lifetime raises the possibility of at least partly addressing the radiation damage-induced challenges outlined above. The physical origin of a dose-rate effect has previously been attributed to the rate of diffusion of free radicals through the crystal lattice, quenching of radicals by solvent molecules and free-radical recombination, although a model relating these effects to the diffracting power of the crystal has until now remained elusive. In order to shed light on the mechanisms of radiation damage at room temperature, we now report data collected at significantly higher dose rates and with the diffracting power sampled with increased temporal resolution.

Despite the use of a state-of-the-art PILATUS2 6M detector (Dectris, Baden, Switzerland), the experiments previously reported were limited to a minimum exposure time of 40 ms (maximum 25 frames per second; fps). A firmware upgrade to the I24 6M detector now allows data collection at 200 fps (minimum exposure time 5 ms; dead time 2.3 ms) when reading out a subarea of the detector, and recently a new generation of PILATUS detectors have become available offering increased frame rates and a reduced dead time. The PILATUS3 300K detector is able to continuously record data at up to 500 fps (minimum exposure time 2 ms; dead time 0.95 ms), allowing X-ray beam-induced effects in protein and virus crystals to be followed on much shorter timescales.

In the work described here, we report a significant deviation from previous observations of a simple exponential or linear decay in the diffracting power of crystals at room temperature. Instead, two phases of decay are observed: an initial slow linear decay, or lag phase, which is then followed by a faster exponential decay. The presence of an initial lag phase truly raises the prospect of collecting largely complete data sets from room-temperature microcrystals. We show that data of comparable quality to those collected from cryocooled crystals can be collected from room-temperature crystals at high dose and frame rates. A macroscopic four-state model outlining the physical origin of this lag phase is reintroduced and discussed.

## 2. Sample preparation and data collection   

### 2.1. Sample preparation   

*Bovine enterovirus* serotype 2 (BEV 2) crystals of ∼30 × 30 × 30 µm in size were grown in space group *F*23, with unit-cell parameters *a* = *b* = *c* ≃ 436.6 Å, in 1.5 *M* ammonium sulfate, 0.1 *M* bis-tris propane pH 7.0 using the sitting-drop method (Walter *et al.*, 2005 ▶). Thaumatin was purchased from Sigma (product No. T7638) and was dissolved in double-distilled H_2_O to a concentration of 40 mg ml^−1^. 4 µl protein solution was mixed with 2 µl reservoir solution consisting of 50 m*M* ADA pH 6.8, 0.6 *M* potassium/sodium tartrate, 20% glycerol, resulting in crystals of approximately 50 × 50 × 50 µm. *Foot and mouth disease virus* (FMDV) A22-wt crystals with average dimensions of 50 × 50 × 5 µm were grown in space group *I*222, with unit-cell parameters *a* = 328, *b* = 341, *c* = 363 Å, in 4 *M* ammonium acetate, 100 m*M* bis-tris propane pH 7.0 using the sitting-drop method (Porta *et al.*, 2013 ▶).

### 2.2. Data collection   

Diffraction data were collected on beamline I24 at Diamond Light Source using a beam size of 10 × 10 or 20 × 20 µm (FWHM of Gaussian profile) at a temperature of 295 K. Data were collected with X-rays of energy 12.8 keV with a flux of 1.4 × 10^12^ photons s^−1^ in the unattenuated beam. Data were also collected at 9.2 keV with an incident flux of 3.2 × 10^12^ photons s^−1^. This allowed dose rates of up to 4.8 MGy s^−1^ (12.8 keV) and 25 MGy s^−1^ (9.2 keV) to be realised. The flux at the sample position was measured using a silicon PIN diode and was calculated as described by Owen *et al.* (2009 ▶). The estimated error in calculation of flux in this manner is <5%. The dose in MGy absorbed by each crystal during a data set was determined using *RADDOSE* (Paithankar *et al.*, 2009 ▶).

Unless otherwise stated, diffraction images were pseudo-stills with an oscillation range of 1 mdeg per image. A continuous rotation, rather than repeated still images, was collected from each crystal as 1 mdeg was the minimum rotation range permitted by the data-collection software. While this method of tracking the diffracting power has the advantage of avoiding the introduction of new material into the beam, it is susceptible to increases in mosaic spread and cell-dimension changes triggered by radiation damage. These changes have the potential to alter reflection centroids and widths and thus introduce new reflections to the image or remove previously observed reflections. In between these extremes we would expect to observe increases and decreases in individual reflections. One of the assumptions that we make is that Bragg spot intensity variations owing to the cell and mosaic effects outlined above will average out over the several hundred recorded reflections in each diffraction image. This assumption is justified in part by the results obtained: data collected from multiple crystals in random orientations exhibit the same trend in intensity decay, with no strong variation in diffracting power between sequential images as would be expected if variation in the unit cell and mosaicity resulted in significant changes to the total diffracting power. Furthermore, *XDS* (Kabsch, 2010 ▶; details of the data analysis are given in §[Sec sec2.3]2.3) showed the variation in unit-cell dimensions to be typically less than 0.5% over the duration of a data set, demonstrating that any contribution to intensity variation arising from changes in unit-cell size is small.

Diffraction data were recorded using two different area detectors: a PILATUS2 6M-F (P2) and a PILATUS3 300K (P3). A software upgrade to the P2 detector allowed the central two modules to be read out at up to 200 fps (minimum exposure time 5 ms). The dead time of this detector is 2.3 ms and the maximum count rate is 2.4 × 10^6^ counts per pixel per second. The P3 300K detector is able to continuously record data at up to 500 fps (minimum exposure time 2 ms, dead time 0.95 ms, maximum count rate >12 × 10^6^ counts per pixel per second). The P3 detector also differs from the P2 detector in that instant retrigger of the counter means that the dead time of the detector is reduced to the duration of a single interaction (Loeliger *et al.*, 2012 ▶).

### 2.3. Data analysis   

X-ray diffraction data were analysed in two ways. Firstly, data were analysed using *LABELIT* (Sauter & Poon, 2010 ▶), using the program *DISTL* to determine the integrated signal strength given in counted photons above the local background of all Bragg candidates. This quantity was calculated on a per-image basis and was defined as the diffracting power of the crystal. Data were also integrated using *XDS* (Kabsch, 2010 ▶) with the diffracting power defined as the sum total of *I*/σ(*I*) over all indexed reflections on the diffraction image. The trend in intensity decay observed was independent of the analysis method used, although we found that the *XDS* approach provided smoother decay curves for weak data.

### 2.4. Intensity decay model   

Sygusch & Allaire (1988 ▶) proposed a model of sequential radiation damage developed from that described by Blake & Phillips (1962 ▶). The Blake and Phillips model postulates that an irradiated protein crystal is made up of three fractions, unchanged, damaged and destroyed, the first two of which contribute to the observed diffraction pattern. This three-state model predicts a simple exponential decay in the diffracting power of a crystal. The Sygusch and Allaire four-state model includes an additional state which also contributes to the observed diffracting power. This state was defined as being characterized by protein that has undergone a number of ionization events or suffered site-specific damage but is broadly unchanged in terms of its scattering power with respect to the unchanged state. In this four-state model, radiation damage follows three steps:
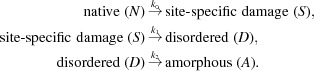



For clarity, we use here the nonclementure *N*, *S*, *D* and *A* to refer to the four states. The contribution of the *N*, *S* and *D* states to the observed diffraction pattern varies as a function of absorbed dose *d*, *i.e.*.
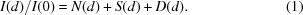
The contribution of *D* is resolution-dependent, but when tracking the total diffracting power of each crystal this dependence can be neglected. Solution of the rate equations derived from this model allows the diffracting power of a crystal to be predicted as a function of absorbed dose. A solution can be determined in two regimes: when *d* < 1/*k*_0_′ and when *d* > 1/*k*_0_′, where *k*_0_′ = *k*_0_ *I*_0_. Initial analysis of intensity-decay profiles indicated that they are best described by the model in the case when *k*_1_ = *k*_2_ ≠ 0 in both of these regimes. The intensity-decay curve is thus described by only two variables *k*_0_ and *k*_1_. In the first regime, when the absorbed dose *d* is less than 1/*k*_0_′, Sygusch and Allaire showed that the diffracting power of a crystal varies as
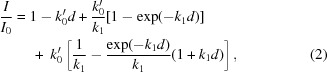
and in the second regime when *d* > 1/*k*_0_′ and *k*_1_ = *k*_2_ as 
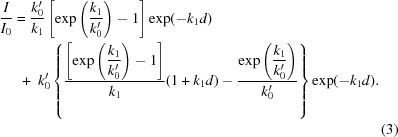
The diffracting power of each crystal as a function of dose was fitted with a dose-response curve of the form (2) in the low-dose region (typically when *I*/*I*_0_ > 0.5) and of the form (3) in the high-dose region.

**Figure 1:**
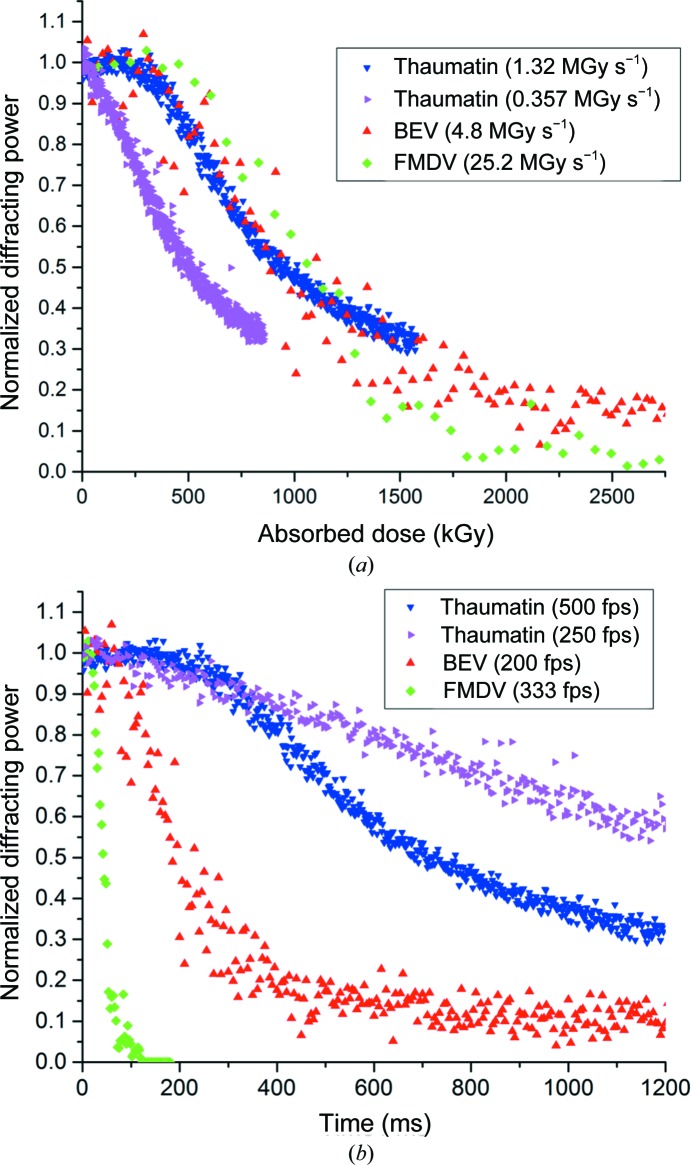
Intensity decay of BEV 2, FMDV and thaumatin single crystals as a function of absorbed dose (*a*) and time (*b*). In contrast to previous observations, a two-phase decay can be seen. The BEV and thaumatin data were collected at an X-ray energy of 12.8 keV and the FMDV data were collected at 9.2 keV. The thaumatin and FMDV data were collected using the P3 detector, and those of BEV with a region of interest of a P2 detector.

The model predicts that the diffracting power tends to zero at high absorbed doses. However, in practice, using the background-subtraction models in the software we used, even images showing no diffraction spots appear to possess nonzero diffracting power, presumably owing to imperfect modelling of the diffuse scatter present on the detector. At very low count levels, the background exhibits strong pixel-to-pixel variation; thus, calculation of the Bragg intensity above background may be overestimated when the diffracting power of the crystal is low. A nonzero diffracting power in the absence of observable spots is best illustrated by the BEV data shown in Fig. 1 ▶: at doses above 2 MGy (times of >500 ms) the diffracting power remains constant at ∼0.15. In this dose and time domain no Bragg spots are visible on the diffraction images and the diffracting power no longer changes as a function of absorbed dose. This baseline intensity of 0.15 must thus be subtracted from the diffracting power if the model is to describe the data well. Changing the threshold for the minimum number of counts in a spot reduces this value at the expense of causing weak high-resolution reflections at the start of the data set to be ignored. The low mosaicity of room-temperature crystals also has an effect: Bragg spots are often 1–2 pixels in size, so the threshold on the minimum number of pixels in a spot must also be set at a low value contributing to integration of background. Owing to this need to both rescale and normalize the data, analysis of the decay of all crystals described in this paper has been performed twice: firstly on normalized and scaled data using the Sygusch and Allaire decay functions presented above and secondly on raw data using a dose-response curve as described below.

Intensity variation as a function of dose was also fitted with a standard dose-response curve of the form
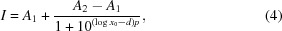
where *A*_2_ represents the upper asymptote (*i.e.* the diffracting power at zero dose), *A*_1_ represents the lower asymptote (the final diffracting power), log*x*_0_ the midpoint of the function, *p* the Hill slope and *d* the absorbed dose using *OriginPro* (OriginLab). This function was chosen as the standard form of the dose-response inhibition curve, *i.e.* a slow initial decay followed by a rapid decay, closely resembles the nonlinear decays in diffracting power observed. A crystal lifetime was defined as the dose when the diffracting power fell to 85% of its initial value, as historically this was seen as the point at which room-temperature data collection should be halted (Blundell & Johnson, 1976 ▶).

## 3. Results   

The intensity decay of three crystal types as a function of absorbed dose is shown in Fig. 1 ▶(*a*). At high dose rates (>1 MGy s^−1^) a clear lag phase lasting ∼500 kGy is visible. The dependence of the lag phase on the dose rate is illustrated by the contrasting intensity decay of thaumatin at incident dose rates of 1.32 and 0.36 MGy s^−1^. The data from FMDV crystals were collected at a lower energy of 9.2 keV at a significantly higher dose rate of 25.2 MGy s^−1^, but do not show a significantly increased lag phase in comparison to the thaumatin and BEV data (all collected at 12.8 keV). Rate constants obtained by fitting Sygusch–Allaire intensity decays are given in Table 1 ▶. Fig. 1 ▶(*b*) shows the same intensity decays as a function of time: in contrast to Fig. 1 ▶(*a*) no correlation is observed between different crystal types. This lack of correlation in the time domain is true for both the duration of the lag phase and the rate of the subsequent faster decay. Thaumatin and FMDV data were recorded using the P3 detector. The BEV 2 data were recorded using the P2 detector.

In all of the experiments described here, the maximum observed count rates were well below the maximum count rate recordable by the detector. The maximum count rates (counts per second; cps) recorded for each crystal type were: BEV 2 (P2), 0.304 Mcps (12.5% of the maximum); thaumatin (P3), 5.35 Mcps (44% of the maximum); FMDV, 1.2 Mcps (9.9% of the maximum). Note that all of the above refer to the single most intense spot within a data set: average spot intensities are an order of magnitude lower than this. For example, in the thaumatin data set, in which a maximum count rate of 5.35 Mcps was recorded, the maximum count rate in the second most intense spot is 2.55 Mcps (21% of the maximum). It is clear from this that the experiments are well within the valid count rate ranges of the detectors used.

**Figure 2:**
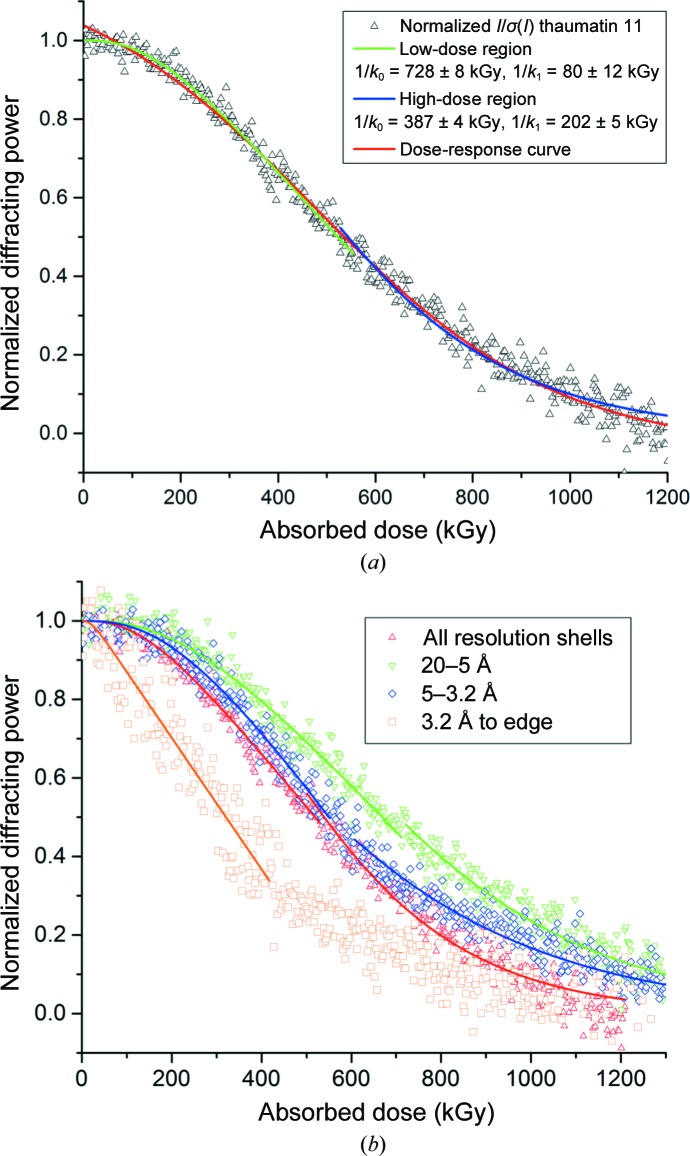
(*a*) A typical intensity decay with (2) (low-dose region) and (3) (high-dose region) and a dose-response curve (4) fitted to the data. The total duration of the experiment is <1 s and data collected from a thaumatin crystal are shown (thaumatin 4 in Supplementary Table S1). The transition from the low-dose region is at approximately 600 kGy (∼400 ms). (*b*) shows data from the same crystal split into resolution bins with (2) and (3) fitted. No fit is shown for the highest resolution shell high-dose region, as the absence/short duration of a lag phase caused the fit to fail: this is discussed in the text. Rate constants for the fits are given in Table 2 ▶.

Fig. 2 ▶(*a*)^1^ shows the intensity decay of a thaumatin crystal with a dose-response curve (4) and Sygusch–Allaire low-dose and high-dose domain (equations 2 and 3) curves fitted. (2) was fitted in the region *I*/*I*_0_ > 0.5 and (3) in the region *I*/*I*_0_ < 0.5. Greater agreement between the rate constants can be obtained by fitting (3) to the region where the absorbed dose *d* is more than or equal to the value of (1/*k*_0_) obtained from (2). If this is performed, then in the high-dose region 1/*k*_0_ and 1/*k*_1_ become 517 ± 25 and 174 ± 12 kGy, respectively. Fig. 2 ▶(*b*) illustrates the variation in the rate of intensity decay for different resolution shells. Rate constants for each fit are given in Table 2 ▶. For the highest resolution shell it was not possible to fit (3) to the data, presumably because the lag phase becomes small compared with the duration of an image.

**Figure 3:**
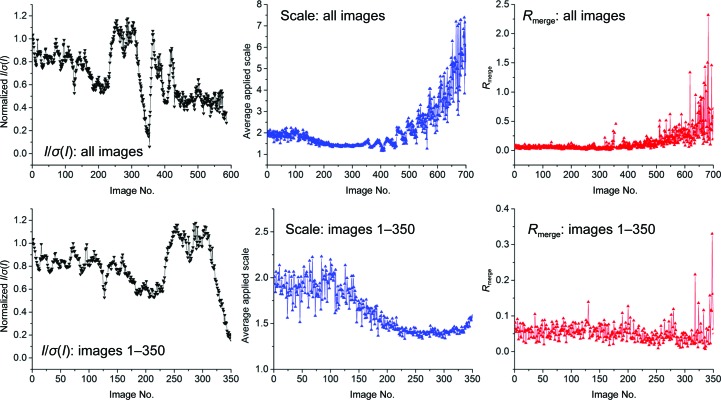
Intensity decay and scaling statistics from a room-temperature lysozyme crystal. Data were collected at a high dose rate, resulting in rapid decay of the crystal. The top row illustrates data collected over 700 images (total exposure time 1.4 s, total absorbed dose 2.35 MGy). The bottom row shows data from the first 350 images of the data set (total exposure time 0.7 s, total absorbed dose 1.17 MGy). Scaling statistics for both data sets are shown in Table 3 ▶.

The presence of a lag phase raises the question of how this can be usefully exploited in room-temperature MX. In order to assess the possibility of exploiting the lag phase, data were collected from a room-temperature lysozyme crystal at 500 fps using the unattenuated beam at I24. For this experiment the beamsize was increased to 50 × 50 µm to match the crystal size and an oscillation angle of 0.1° per image was used. Scaling statistics for 700 images (total exposure time 1.4 s, cumulative absorbed dose 2.35 MGy) are shown in the top row of Fig. 3 ▶ and Table 3 ▶. An effect of crystal rotation is the introduction of artefacts into the diffracting power as a function of frame number. What is clear is the lack of variation in scales and *R*_merge_ for the first half of the data set. Scaling the first 350 images (bottom row of Fig. 3 ▶; Table 2 ▶) results in a data set indistinguishable in quality from one collected at 100 K as judged by the scaling metrics detailed above and the statistics in Table 3 ▶.

**Figure 4:**
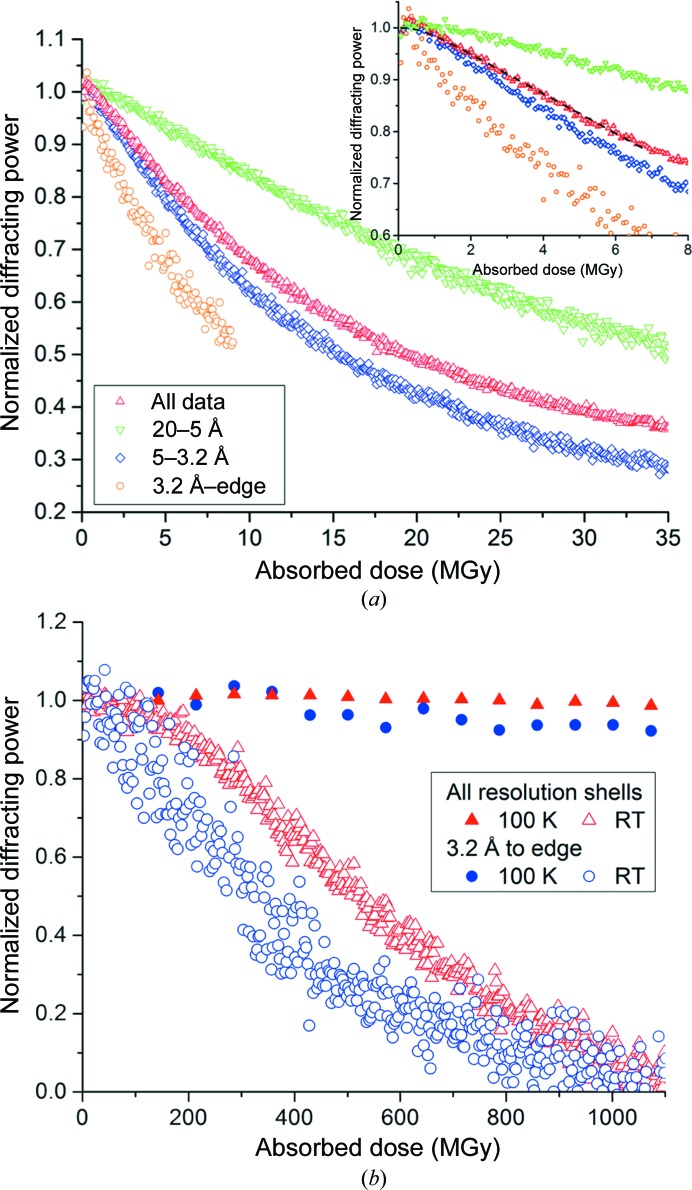
Resolution-dependent intensity decay of a thaumatin crystal at 100 K (*a*); data were collected at a dose rate of ∼480 kGy s^−1^. In the low-dose region a departure from a simple exponential decay can be seen (inset). (2) is fitted to the low-dose region for the total diffracting power (dashed line). Comparison of the decay of the cryocooled crystal in the top section of the figure with the room-temperature thaumatin crystal shown in Fig. 2 ▶ (dose rate 1.32 MGy s^−1^) shows a similar rate of decay at 100 K and room temperature at very low doses (*b*).

Fig. 4 ▶ compares further the contrast between room temperature and 100 K. Fig. 4 ▶(*a*) shows the resolution-dependent change in diffracting power of a thaumatin crystal as a function of absorbed dose at 100 K. The absence of a dose-rate effect at 100 K (Sliz *et al.*, 2003 ▶) means that the difference in dose rate between the samples should not affect the comparison despite the differing dose rates used. While a lag phase is not immediately apparent, deviation from a linear or exponential decay is visible at low doses (Fig. 4 ▶ *a*, inset). This is most clearly visible for the low-resolution data (green triangles), but is also apparent for ‘all data’ (red triangles). If an exponential decay is fitted then the residual is approximately constant over the whole data range except at very low doses (<1 MGy), when it increases sharply, indicating that an exponential decay function describes this region relatively poorly. A Sygusch–Allaire intensity decay (2) has been fitted to the low-dose domain of the total intensity decay with 1/*k*_0_ = 26.2 ± 10 kGy and 1/*k*_1_ = 336 ± 6 kGy, and while this shows some deviation at doses of <1 MGy, it provides a better description of the observed decay than a simple exponential decay function.

Fig. 4 ▶(*b*) overlays data from Figs. 2 ▶(*b*) (room temperature) and 4 ▶(*a*) (100 K). The dose required to halve the diffracting power of the crystal (all resolution shells) at 100 K is 19.4 MGy, whereas the dose required to halve the diffracting power of the room-temperature crystal is 525 kGy, a factor of ∼37 less. The short duration of the room-temperature lag phase coupled with frame-to-frame variations in the diffracting power make it difficult to determine what the crystal lifetime would be if this slow rate of decay were to be extended by, for example, collecting at higher dose rates or reducing the temperature. Based on a linear fit to data within the room-temperature lag phase (the first 50 data points), if the lag phase could be extended further, then the dose required to halve the diffracting power at room temperature would be ∼1.8 MGy, a factor of only 7.5 less than the lifetime at 100 K.

## 4. Discussion   

Following observation of X-ray-induced decay of myoglobin crystals, Blake & Phillips (1962 ▶) proposed that irradiated protein crystals are made up of a linear combination of three states: an unchanged fraction *A*_1_, a disordered fraction *A*_2_ and an amorphous fraction *A*_3_. The fractions *A*_1_ and *A*_2_ contribute to the observed diffraction pattern such that the diffracting power of the crystal falls off in the form
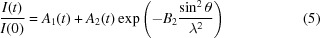
as a function of time (or dose). An intensity decay of this form is unable to describe the observations detailed in §[Sec sec3]3 of a lag phase followed by a rapid exponential decay. The limiting aspect of the model is that only two states can contribute to the diffracting power of the crystal: this limitation also applies to developments of the three-state model (for example, Hendrickson, 1976 ▶). The Sygusch and Allaire model described in §[Sec sec2.4]2.4 postulates that upon irradiation the diffracting power of a protein crystal can be described by sequential progression through four states [native (*N*), site-specific (*S*), disordered (*D*) and amorphous (*A*)]. In this model, three states (*N*, *S* and *D*) contribute to the diffracting power.

The four-state model predicts nonlinear and non-exponential intensity decay, in agreement with the observed intensity decays presented here. The diffraction data presented here are best described by the *NSDA* model in the case when *k*_1_ (*S*→*D*) is equal to *k*_2_ (*D*→*A*). Provided that the timescale (1/*k*_0_) of the transition from native to site-specific (*N*→*S*) within the sample is slow compared with the experiment, a clearly resolvable delay in the apparent onset of radiation damage results. This is the lag phase observed in Figs. 1 ▶ and 2 ▶. A lag phase was first observed in a Laue experiment through the use of a streak camera by Moffat *et al.* (1986 ▶), but to our knowledge was not pursued. This effect has not been observed on a monochromatic source as until now the frame rate at which detectors can operate has been significantly larger than the timescale of this transition. Prophetically, Sygusch and Allaire remarked ‘this delay could be advantageously exploited by efficient two-dimensional detection systems’, as is the case here with a detector capable of collecting 500 frames per second.

**Figure 5:**
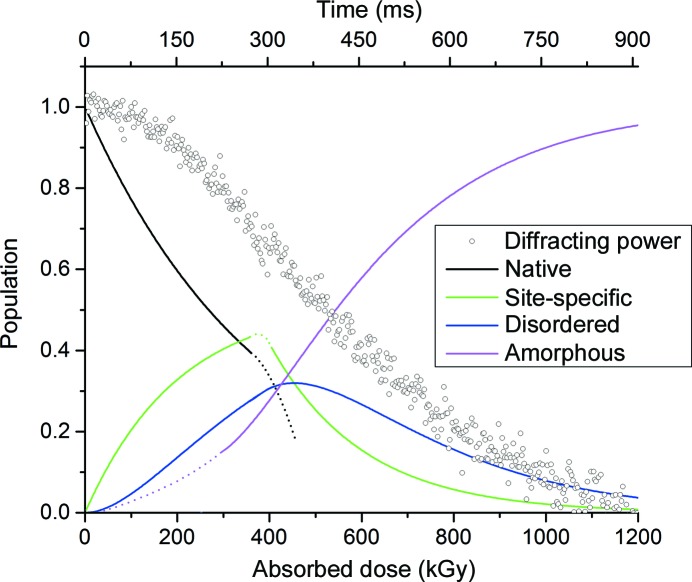
Populations of the native, site-specific, disordered and amorphous fractions of the crystal as a function of absorbed dose as predicted using the *NSDA* model. Populations were calculated using rate constants determined from the fits to the data shown in Fig. 2 ▶(*a*). Note that in the crossover region between the low-dose and high-dose regions (∼300–500 kGy) the populations are an approximation: these regions are shown as dashed lines. The diffracting power of the crystal is overlaid.

Following determination of the rate constants *k*_0_ and *k*_1_, the population of each fraction of the crystal in both regions can be plotted as a function of absorbed dose. This is shown for the thaumatin crystal decay shown in Fig. 2 ▶ in Fig. 5 ▶. Fig. 5 ▶ highlights the result of the sequential damage process on the observed intensity decay. At low doses the intensity decay is slow while the undamaged fraction of the crystal is converted to the dose-dependent state *S* characterized by site-specific rather than global damage. Subsequent to this significant disruption to the lattice occurs, represented by the increasing population of the states *D* and *A*, and the diffracting power follows an exponential-like decay. The diffracting power mirrors the fraction of the crystal in the amorphous state *A.*

Comparison of room-temperature and cryogenic rates of decay (Fig. 4 ▶ *b*) shows that the temperature-dependence of *k*_0_, which represents the rate at which the population of the site-specific fraction of the crystal increases, is large. The change in 1/*k*_0_ observed between thaumatin held at room temperature (417 kGy) and 100 K (26.2 MGy) is a factor of 63. This is remarkably similar to the factor of ∼70 increase in lifetime previously reported in the literature (Southworth-Davies *et al.*, 2008 ▶), although it should be noted that the accompanying change in *k*_1_ (a factor of 1.8) also results in a change in the rate of intensity decay. The large decrease in *k*_0_ at low temperatures is a result of the reduced mobility of free radicals, particularly hydroxyl (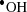), at 100 K, resulting in a greatly reduced rate of site-specific damage. The presence of a dose-rate effect at room temperature can, as previously postulated, be attributed to a number of effects: the rate of diffusion of free radicals through the crystal lattice, the quenching of radicals by solvent molecules and free-radical recombination. While it is not yet possible to say which of these dominates, the net result is reflected by a change in the rate constant *k*_0_.

It should be noted the increase in 1/*k*_0_ at low temperatures does not imply that cryocooling completely mitigates rapid site-specific damage, or that structures determined at 100 K are free of damage artefacts. Numerous studies in the literature, in particular those making use of complementary methods, have reported the extremely rapid onset of site-specific damage even at temperatures below 130 K. Several approaches, including XAS (Oliéric *et al.*, 2007 ▶), UV–Vis (Beitlich *et al.*, 2007 ▶) and EPR plus UV–Vis (Sutton *et al.*, 2013 ▶), have reported lifetimes of less than 1 MGy in comparison to lifetimes of >20 MGy based on intensity decay. For room-temperature data collection it is a concern that data sets, or partial data sets, predominantly represent a damaged rather than the native structure. From Fig. 5 ▶ it can be seen that the estimated population of the site-specific state does not become greater than the population of the native state until *I*/*I*_0_ ≃ 0.70.

Beam-induced heating is a potentially limiting problem if the lag phase is to be fully exploited. A calculation based on equation 8 in Warkentin *et al.* (2012 ▶) suggests a steady-state temperature difference of 32.5 K for the high dose-rate (4.8 MGy s^−1^) BEV data described here. As the crystals used were larger than the beam size, the so-called ‘fin effect’ acts to minimize any temperature increase within the irradiated volume. Measurements by Snell *et al.* (2007 ▶) of the temperature change induced by X-rays within glass beads suggests that a steady state is not reached for ∼5 s, although approximately half of the final temperature rise is realised within ∼1 s of exposure to X-rays. For samples smaller than the 1 and 2 mm diameter glass beads used by Snell and coworkers, it can be expected that temperature changes occur on faster timescales. However, given the short duration of the experiments described here (<1 s), we believe the steady-state figure of 33 K provides an upper bound for the potential temperature rise in the typical lifetime of crystals (<0.5 s). Using the same model, a greater temperature rise (a factor of 5.3) would be expected for the FMDV data shown in Fig. 1 ▶. Observation of crystals during exposure to X-rays at these dose rates (∼25 MGy s^−1^) suggests that a major temperature rise is not realised, since no obvious signs of boiling are observed. Nevertheless, at higher temperatures rates of diffusion and reaction rates are increased and thus beam-induced heating may well result in a narrowing of the lag-phase window. We cannot rule out that this may have a significant effect on some of the results presented here, and beam-induced heating may prevent full exploitation of the lag phase when samples are subjected to higher fluxes, which will lead to larger and faster temperature changes. The use of humidifier devices providing a continuous flow of gas at, or just below, room temperature may partially alleviate heating effects, but proper quantification of this is required.

**Figure 6:**
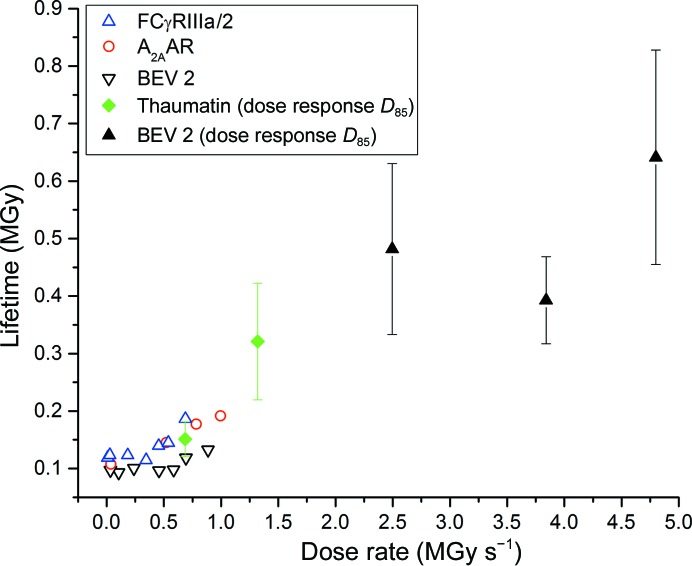
Dose required to reach 85% of the initial diffracting power for BEV 2 and thaumatin crystals overlaid on the lifetimes reported in Owen *et al.* (2012 ▶). The new points show the mean lifetimes of 11 thaumatin crystals and 18 BEV 2 crystals and are also shown in Supplementary Fig. S1.

Fig. 6 ▶ compares the data in this study with the lifetimes reported in Owen *et al.* (2012 ▶). Consistent with these earlier results, we here observe a dose-rate dependence of crystal lifetime, with increased lifetimes at higher dose rates. The origin of a dose-rate effect was postulated to result from three effects: free-radical diffusion, solvent quenching and recombination. The work here provides evidence for a macroscopic model explaining the form of the X-ray-induced decay in the diffracting power of crystals but does not directly shed light onto the processes and species causing this decay. The model does provide approximate dose scales for when diffusion, quenching and recombination occur as these might be considered to be most significant before the transition from the native to the site-specific state (*N*→*S*) is complete.

Despite the relatively small window before crystal destruction, it is possible to collect high-quality room-temperature diffraction data at high dose and frame rates (Table 2 ▶). Despite a dead time of 0.95 ms per 2 ms exposure, good scaling statistics are obtained. This is presumably owing in part to the mosaicity of the crystal (reported to be 0.36° by *XDS*) being significantly larger than the oscillation angle of 0.1° per image. While the data in the example shown are incomplete, the possibility of collecting significant wedges of data from room-temperature microcrystals significantly increases the scope of *in situ* MX for, for example, high-throughput rapid ligand-screening experiments, removing the need to mount and cryocool samples.

It is important to note variation between different crystal types: this highlights the usefulness of the information that can be provided by exploratory data sets (Krojer & von Delft, 2011 ▶). At room temperature, we suggest that the optimal approach to confirm what the lag phase is for a particular crystal type with the flux density at the beamline being used would be the collection of stills, or very small oscillation, images from a sacrificial crystal. Data collection using still images minimizes any contribution from crystal rotation as observed in Fig. 3 ▶. Nonetheless, some variation between crystals should still be expected.

In conclusion, the data presented here show a clear departure from a linear or an exponential decay in the diffracting power of room-temperature protein crystals. The presence of a lag phase at low doses can be explained through consideration of a sequential four-state model, the first three states of which contribute to the observed diffraction pattern. Comparison of data from crystals held at cryogenic and room temperatures illustrates the applicability of the model to both cases. The ability to rapidly collect diffraction data from room-temperature crystals within the lag phase raises the possibility of significantly reducing the number of crystals required for room-temperature structure solution.

## Supplementary Material

Supporting Information.. DOI: 10.1107/S1399004714005379/wa5063sup1.pdf

## Figures and Tables

**Table 1:** Rate constants extracted from fits of (2) and (3) to the intensity decays of the crystals shown in Fig. 1 ▶(*a*) The dose required to reduce the diffracting power to 85% of the initial diffracting power (*D*_85_) as determined from a fit of a dose-response curve (4) is also given. A fit of (2) and (3) to the FMDV data is not appropriate owing to the small number of data points in each region. *D*_85_ for the FMDV data is 660 kGy.

		Low-dose region (2)		High-dose region (3)		
Crystal	Dose rate (MGy s^−1^)	1/*k*_0_ (kGy)	1/*k*_1_ (kGy)	1/*k*_0_ (kGy)	1/*k*_1_ (kGy)	*D*_85_ (kGy)
Thaumatin	0.357	632 ± 1	107 ± 2	171 ± 26	155 ± 10	118
Thaumatin	1.32	728 ± 8	80 ± 12	387 ± 4	202 ± 5	418
BEV	4.8	722 ± 8	263 ± 31	346 ± 11	411 ± 3	400

**Table 2:** Rate constants extracted from fits of (2) and (3) to the intensity decays of the thaumatin crystal shown in Fig. 2 ▶(*b*) The numbers of reflections in each resolution bin are 61 (20–5 Å), 63 (5–3.2 Å) and 70 (3.2 Å to edge).

	Low-dose region (2)		High-dose region (3)	
Resolution range	1/*k*_0_ (kGy)	1/*k*_1_ (kGy)	1/*k*_0_ (kGy)	1/*k*_1_ (kGy)
All	728 ± 8	80 ± 12	387 ± 4	202 ± 5
20–5 Å	713 ± 2	154 ± 40	501 ± 3	262 ± 3
5–3.2 Å	633 ± 2	121 ± 80	148 ± 12	286 ± 2
3.2 Å–edge	598 ± 2	111 ± 5	—	—

**Table 3:** Data-quality metrics for the data illustrated in Fig. 3 ▶ In addition to metrics for each resolution shell, the resolution at which the correlation coefficient (Karplus & Diederichs, 2012 ▶) between random half sets (CC_1/2_) falls to 0.5 is given. Data were recorded using a beamsize of 50 × 50 µm at 500 fps (exposure time 2 ms). An oscillation of 0.1° per image was used, resulting in data collection at an angular velocity of 50° s^−1^. The total angular range covered is 70° (all images) and 35° (the first 350 images).

	All images			First 350 images only		
	Overall	Inner shell	Outer shell	Overall	Inner shell	Outer shell
Low-resolution limit (Å)	55.4	55.4	2.33	55.4	55.4	2.33
High-resolution limit (Å)	2.25	8.7	2.25	2.25	8.7	2.25
*R*_merge_	0.110	0.032	0.605	0.027	0.018	0.103
*R*_meas_	0.141	0.039	0.852	0.037	0.023	0.145
Correlation coefficient	0.978	0.997	0.159	0.998	0.998	0.964
Total No. of observations	8113	229	487	4105	137	248
Mean *I*/σ(*I*)	7.3	15.7	2.4	12.6	38.3	3.0
Completeness (%)	54.3	66.7	49.8	46.2	35.2	46.5
CC	0.978	0.997	0.159	0.998	0.998	0.964
Resolution (CC_1/2_ = 0.5) (Å)	2.45			2.33		

## Footnotes

1 Supporting information has been deposited in the IUCr electronic archive (Reference: WA5063).
