# Supplementary material for: Exploiting fast detectors to enter a new dimension in room-temperature crystallography
Source: Acta Crystallogr D Biol Crystallogr. 2014 Apr 26;70(Pt 5):1248–56. doi: 10.1107/S1399004714005379 (PMC4014120; doi:10.1107/S1399004714005379)
Supplement: Supplementary file 1 [file d-70-01248-sup1.pdf]

Acta Crystallographica Section D

Volume 70 (2014)

Supporting information for article:

Exploiting fast detectors to enter a new dimension in room-temperature crystallography

Robin L. Owen, Neil Paterson, Danny Axford, Jun Aishima, Clemens Schulze-Briese, Jingshan Ren, Elizabeth E. Fry, David I. Stuart and Gwyndaf Evans

Table S1 Dose rates used and observed crystal lifetimes for the thaumatin and BEV 2 crystals. The lifetime is given as the dose required to reduce the diffracting power to 85% of the initial diffracting power, D_{85}. The mean for each crystal type and dose-rate is shown in figure 6, the mean lifetimes of each crystal type is shown as a function of dose-rate in figure S1.

Crystal	Dose Rate / kGy s^{-1}	D_{85} / kGy
Thaumatin 1	687	144
Thaumatin 2	687	192
Thaumatin 3	687	150
Thaumatin 4	1320	418
Thaumatin 5	1320	249
Thaumatin 6	1320	276
Thaumatin 7	1320	303
Thaumatin 8	1320	348
Thaumatin 9	1320	177
Thaumatin 10	1320	475
Thaumatin 11	1320	386
BEV 1	2494	422
BEV 2	2494	328
BEV 3	2494	376
BEV 4	2494	649
BEV 5	2494	685
BEV 6	2494	431
BEV 7	3840	321
BEV 8	3840	425
BEV 9	3840	393
BEV 10	3840	322
BEV 11	3840	502
BEV 12	3840	365
BEV 13	4800	863
BEV 14	4800	873
BEV 15	4800	531
BEV 16	4800	711
BEV 17	4800	537
BEV 18	4800	608

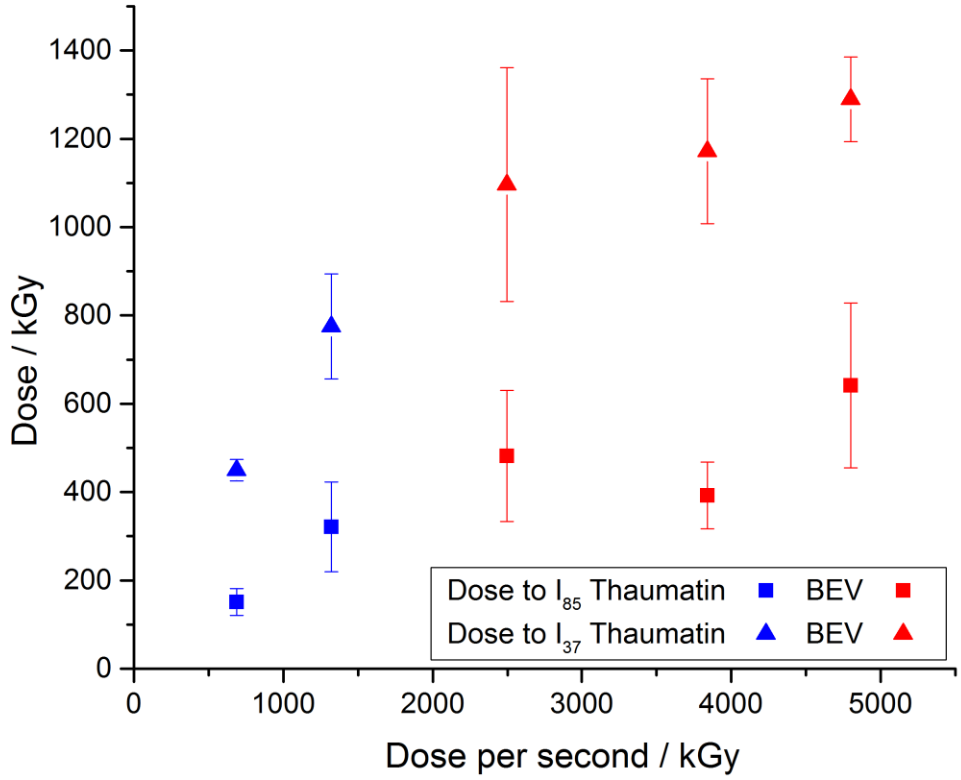

Figure S1 Lifetime of BEV and thaumatin crystals as a function of dose-rate determined using a fitted dose-response curve. Two lifetimes are shown: the dose required to reduce the diffracting power of crystals to 85% of the initial diffracting power, and also the dose required to reduce the diffracting power of crystals to 37% (*i.e.* approximately equivalent the decay constant of an exponential function). These data are shown overlaid on data from Owen *et al*. (2012) in figure 6.
